# Urinary proteome analysis enables assessment of renoprotective treatment in type 2 diabetic patients with microalbuminuria

**DOI:** 10.1186/1471-2369-11-29

**Published:** 2010-11-01

**Authors:** Sten Andersen, Harald Mischak, Petra Zürbig, Hans-Henrik Parving, Peter Rossing

**Affiliations:** 1 Steno Diabetes Centre, Gentofte, Denmark; 2 BHF Glasgow Cardiovascular Research Centre, Glasgow, United Kingdom; 3 Mosaiques diagnostics GmbH, Hannover, Germany; 4 Department of Medical Endocrinology Rigshospitalet, University Hospital of Copenhagen, Copenhagen Denmark; 5 Faculty of Health Sciences, University of Aarhus, Aarhus, Denmark; 6 members of EuroKUP

## Abstract

**Background:**

Previously the angiotensin II receptor blocker Irbesartan has been demonstrated to reduce the risk for progression from microalbuminuria to macroalbuminuria in type 2 diabetic patients. The purpose of this study was to evaluate the effect of treatment with Irbesartan in type 2 diabetic patients with microalbuminuria on the urinary proteome.

**Methods:**

High-resolution capillary-electrophoresis coupled to mass-spectrometry (CE-MS) was used to profile the low-molecular-weight proteome in urine of a subgroup of patients from a two year randomized irbesartan versus placebo therapy trial, which included hypertensive type 2 diabetic patients with microalbuminuria on ongoing antihypertensive medication (IRMA2-substudy).

**Results:**

We demonstrate that the therapy with 300 mg Irbesartan daily over a period of two years results in significant changes of the urinary proteome. Both, a classifier developed previously that consists of urinary peptides indicative of chronic kidney disease, as well as several individual peptides changed significantly after treatment. These changes were not observed in the placebo-treated individuals. Most prominent are changes of urinary collagen fragments associated with progression of diabetic nephropathy, indicating normalization in urinary peptides.

**Conclusion:**

CE-MS analysis of urine enabled identification of peptides as potential surrogate markers for renoprotection in microalbuminuric type 2 diabetic patients, which show persistent improvement after longterm treatment with Irbesartan. The results suggest that a major benefit of treatment by Irbesartan may be improvement of collagen turnover, reduction of fibrosis. They further suggest that urinary proteome analysis could be utilized to assess potential benefit of therapeutic intervention, providing statistically significant results even on a small population.

## Background

At present more than 170 million people worldwide have diabetes and the number is expected to double within the next 20 years mainly due to an epidemic increase in the prevalence of type 2 diabetes [1]. Type 2 diabetes is associated with an increased occurrence of cardiovascular disease and approximately 40% of all diabetic patients are at risk of developing diabetic nephropathy which has become the leading cause of end-stage renal disease (ESRD) in the Western world [2]. Therefore, the early identification and subsequent end-organ protective treatment of all patients at risk for ESRD is of outmost importance. Patients with persistent microalbuminuria [urinary albumin excretion (UAE) between 30 and 300 mg/24 hours] have a 10 to 20 times increased risk of developing diabetic nephropathy as compared to patients with normoalbuminuria [2]. In addition, the occurrence of microalbuminuria is associated with an increased risk of premature death due to cardiovascular disease [3].

Reduction of UAE by blockade of the renin-angiotensin-aldosterone system (RAAS) has emerged as a key treatment goal for both reno- and cardiovascular protection [4,5]. Data from the large clinical "Irbesartan in Patients with type 2 diabetes and Microalbuminuria" (IRMA2) study [6] firmly demonstrated that treatment with the angiotensin II receptor blocker (ARB) Irbesartan, 300 mg once daily, reduces UAE and the risk of progression to overt diabetic nephropathy in hypertensive patients with type 2 diabetes and persistent microalbuminuria. Furthermore, in type 2 diabetic patients with more advanced renal disease, ARBs have been shown to reduce the risk of reaching the combined renal end point of doubling in serum creatinine, ESRD, or death [5,7]. Since 2002, ARBs have consequently been recommended as first-line therapy in hypertensive type 2 diabetic patients with microalbuminuria or overt diabetic nephropathy according to guidelines from the American Diabetes Association [8].

Recently, we and others demonstrated that diabetic nephropathy and chronic renal disease in general are reflected by specific peptides and proteins in urine [9-24], and the human urinary proteome has been extensively investigated to gain insight about disease processes affecting the kidney and the urogenital tract [12,25-28]. Urinary proteins and peptides originate not only from glomerular filtration, but also from tubular secretion, epithelial cells shed from the kidney and urinary tract, secreted exosomes [29], and seminal secretions [30-32]. Thus, in principle, urine is a rich source of biomarkers for a wide range of diseases due to specific changes in its proteome [33-36]. Urine is a preferred body fluid for proteome analysis, as it is quite stable, probably due to the fact that it is "stored" for hours in the bladder, hence proteolytic degradation by endogenous proteases, a major obstacle in proteomics studies focusing on blood [37], may be essentially complete by the time of voiding [38,39]. This also enabled the establishment of human urine reference standard samples [40]. In pilot studies aiming toward differential diagnosis of certain types of CKD we could show that several peptides are differentially excreted in the urine of patients with different chronic kidney diseases compared to healthy individuals [41,42]. An optimized protocol for sample preparation and analysis has been developed, that includes removal of proteins above 25 kDa without significant loss of low-molecular-weight urinary components [43]. Using this protocol, urinary biomarkers enabling differential diagnosis of specific single chronic renal diseases (IgA nephropathy, diabetic nephropathy, and ANCA-associated vasculitis) with good sensitivity and specificity in blinded data-sets could be identified [13,19,21,44]. Employing previously established biomarkers and biomarker patterns as classifiers [19,20], we investigated if a therapeutic benefit of Irbesartan in microalbuminuric type 2 diabetes patients can by displayed by proteomic changes in urine. In addition, we aimed at identifying those peptides that show significant changes upon Irbesartan treatment, as these may reveal further insights into the pathophysiology of disease, and allow assessment of therapeutic efficacy.

## Methods

### Patient characteristics

Spontaneously voided urine samples were collected from type 2 diabetic patients followed at Steno Diabetes Center as a subset of the 'IRMA2' study described previously [45]. The study was in compliance with the Helsinki Declaration and all patients gave written informed consent. The study was approved by the ethics committee of Copenhagen County KA 97015 gms. Samples from all patients included in the study receiving either Irbesartan in a dose of 300 mg once daily or placebo were employed for CE-MS analysis, if samples were available from both, baseline and after two years of treatment. As the effect of a dose of 150 mg once daily was not significant on UAER, in the IRMA2 study, we only used 300 mg daily and compared with placebo. In total, samples from 22 patients (11 irbesartan and 11 placebo) were available. At baseline 2 patients in the placebo and 4 in the irbesartan group were treated with insulin, after 2 years it was 5 and 4. Unchanged throughout the study, 8 patients in the placebo and 6 in the irbesartan group were treated with oral hypoglycemic agents at baseline, 3 patients in each group were treated with a statin at baseline, 8 patients in the placebo and 5 in the irbesartan group were treated with aspirin for cardiovascular protection at baseline. Demographic data of the patients included are shown in **Additional file **1, spreadsheet: 'patient data'.

### Sample preparation

Samples consisted of overnight urines, stored in aliquots at -20°C for 8-12 years, which were prepared essentially as described [46]. A 0.7 mL aliquot was thawed immediately before use and diluted with 0.7 mL 2 M urea, 10 mM NH_4_OH containing 0.02 % SDS. In order to remove high molecular weight polypeptides, samples were filtered using Centrisart ultracentrifugation filter devices (20 kDa molecular weight cut-off; Sartorius, Goettingen, Germany) at 3,000 g until 1.1 mL of filtrate was obtained. Subsequently, filtrate was desalted using PD-10 column (GE Healthcare, Sweden) equilibrated in 0.01% NH_4_OH in HPLC-grade water. Finally, samples were lyophilized and stored at 4°C. This procedure results in an average recovery of sample in the preparation procedure ~85% [21]. Shortly before CE-MS analysis, lyophilisates were resuspended in HPLC-grade water to a final protein concentration of 0.8 μg/μL checked by BCA assay (Interchim, Montlucon, France).

### CE-MS analysis

CE-MS analysis was performed as previously described [37,47]. The limit of detection was ~1 fmol, mass resolution was above 8000 enabling resolution of monoisotopic mass signals for z≤ 6. After charge deconvolution, mass deviation was < 25 ppm for monoisotopic resolution and < 100 ppm for unresolved peaks (z > 6). The analytical precision of the platform was assessed by (a) reproducibility achieved for repeated measurement of the same replicate and (b) by the reproducibility achieved for repeated preparation and measurement of the same urine sample; details on analytical precision were reported recently [21]. To ensure high data consistency, a minimum of 950 peptides/proteins had to be detected with a minimal MS resolution of 8,000 in a minimal migration time interval of 10 minutes.

### Data processing

Mass spectral ion peaks representing identical molecules at different charge states were deconvoluted into single masses using MosaiquesVisu software [48]. Both CE-migration time and ion signal intensity (amplitude) show variability, mostly due to different concentration of ions in the sample, and are consequently normalized. Reference signals of 1770 urinary polypeptides are used for CE-time calibration by local regression. For normalization of analytical and urine dilution variances, MS signal intensities are normalized relative to 29 "housekeeping" peptides generally present in at least 90% of all urine samples with small relative standard deviation. For calibration, local regression is performed [49]. The obtained peak lists characterize each polypeptide by its molecular mass [Da], normalized CE migration time [min] and normalized signal intensity. All detected peptides were deposited, matched, and annotated in a Microsoft SQL database allowing further statistical analysis.

### Data analysis

The datasets were examined either with respect to significant changes in single, predefined peptides and with respect to scoring in biomarker models (see **Additional file **1, spreadsheet: 'classification factor'). These biomarker models consist of 65 or 273 biomarkers respectively, which were previously found to be significantly associates with diabetic nephropathy [19] or chronic kidney disease [20].

For the application of the previously established biomarker patterns, Wilcoxon test (for paired samples) was performed to receive Box-and-Whisker plots and dot-and-line diagrams [50] (MedCalc version 8.1.1.0, MedCalc Software, Belgium, http://www.medcalc.be).

For multiple testing corrections, p-values were corrected using the false discovery rate procedure introduced by Benjamini and Hochberg, [51]. To eliminate sporadic findings, only proteins that were detected in a diagnostic group of patients in at least 50% of samples were considered.

## Results

**Figure 1:**
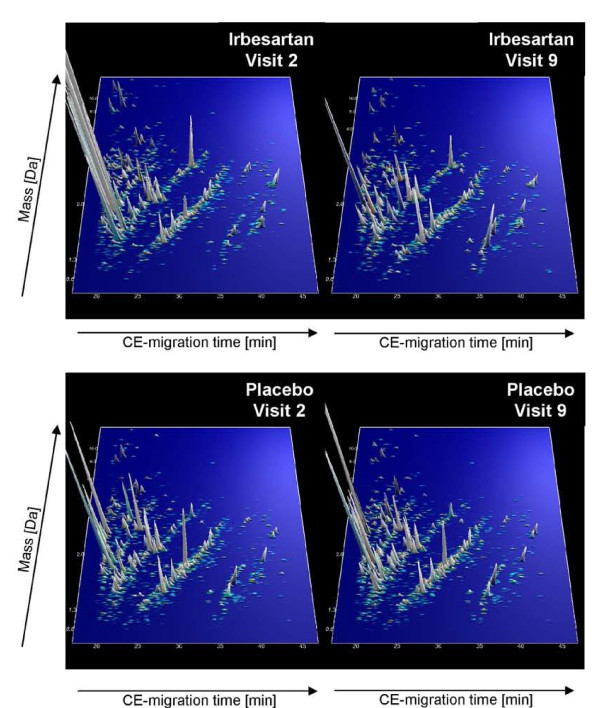
**Polypeptide patterns of patients with diabetes type 2 before and after 2-year treatment (Irbesartan and placebo) examined in the 'IRMA2' study**. Shown are compiled patterns consisting of all samples from each of the four groups. The molecular mass (0.7 to 15 kDa, on a logarithmic scale) is plotted against normalized migration time (17 to 47 min). Signal intensity is encoded by peak height and color.

Samples from 22 patients included in the IRMA2 trial, where urine was collected at baseline before treatment (visit 2) and after two years treatment (visit 9) with Irbesartan or placebo, were analyzed. All available samples were included in the study, and analyzed using CE-MS, no additional specimens that fit the criteria (2 years follow up, placebo or 300 mg Irbesartan daily) are available from the IRMA2 trial. All samples analyzed passed the threshold of the quality control criteria given in the Methods section, no significant deterioration of peptides due to storage could be observed. The data from all analyses are presented in the **Additional file **1. As shown in **figure **1, the compiled data of these 4 groups disclosed first insights into changes of the urinary proteome, where high concentrations of some peptides decreased with Irbesartan intake. To assess the relevance of any proteomics changes with respect to diabetic nephropathy, we applied already established polypeptide patterns onto these data.

First, data from patients that received ARB treatment were evaluated applying a biomarker pattern indicative for diabetic nephropathy [19]. This analysis revealed no significant differences (p = 0.175) between these two groups of patients (visit 2 and visit 9) using Wilcoxon-test for paired samples (data not shown). However, the DN pattern was developed employing samples from diabetes type 1 patients treated with ARB [19], hence may not be applicable for type 2 diabetic patients, and may further be inappropriate to reflect drug-induced changes.

**Figure 2:**
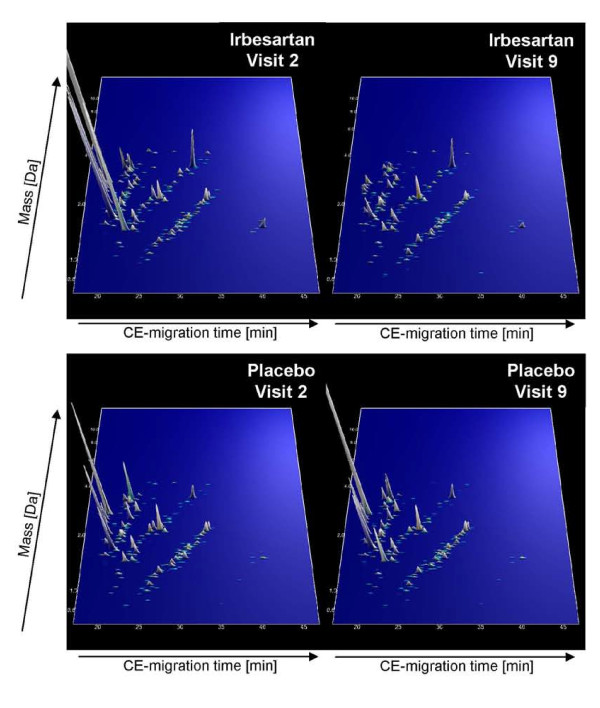
**Peptide patterns of 273 CKD marker used for the proteomic analysis of patients from the 'IRMA2' subgroup**. The compiled data sets of urine samples from patients derived from the 'IRMA2 study' before and after 2-year treatment of Irbesartan (upper panel) as well as placebo (lower panel) are shown. Normalized molecular mass (y-axis) is plotted against normalized CE-migration time (x-axis). The mean signal intensity is represented in 3D-depiction.

**Figure 3:**
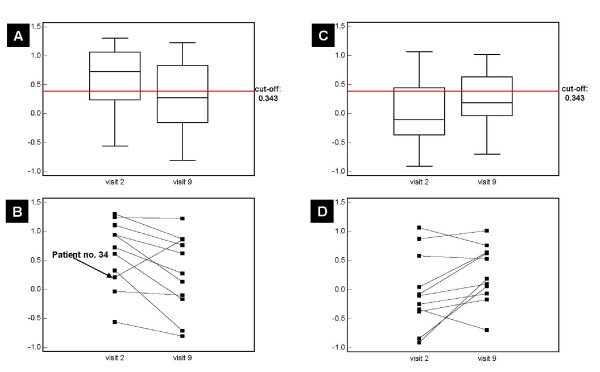
**Classification results of the 'IRMA2' patient samples, classified with the CKD model **[20]. **A) **Box-and-Whisker plot of microalbuminuric patients before (visit 2) and after two years (visit 9) treatment with 300 mg Irbesartan. The red line indicates the cut-off of the CKD model (classification factors above this cut-off are suffering from renal disease). **B) **Dot-and-line diagram of microalbuminuric patients before (visit 2) and after two years (visit 9) treatment with 300 mg Irbesartan. Classification factors of all patients, excepting patient no. 34, declined after Irbesartan intake. **C) **Box-and-Whisker plot of microalbuminuric patients before (visit 2) and after two years (visit 9) treatment with placebo. **D) **Dot-and-line diagram of microalbuminuric patients before (visit 2) and after two years (visit 9) placebo administration.

We therefore also employed a polypeptide pattern indicative of chronic kidney disease (CKD), that consist of 273 known peptides [20] for the classification of the urine samples from the 'IRMA2' study. This model is based on the CE-MS analysis of urine samples from 340 patients with CKD of different etiologies (including focal segmental glomerulosclerosis, membranous glomerulonephritis, minimal change disease, IgA nephropathy, systemic lupus erythematosus, ANCA-associated vasculitis, and diabetic nephropathy) and 550 controls (healthy individuals as well as patients without any evidence for renal diseases). **Figure **2 demonstrates the changes of these 273 peptides of the CKD model before and after treatment with Irbesartan and placebo, respectively. While the peptide pattern of the ARB treated patients is similar to that observed for diabetic nephropathy (compare Figure 1 in [19]) at the beginning of the study (prior treatment), it changed towards higher similarity to normalbuminuric subjects after 2 years of Irbesartan treatment. As depicted in the Box-and-Whisker plot in **figure **3A, this classification resulted in a significant (p = 0.0244) decline of the median classification factor (indicating an improvement of the kidney physiology), which was reduced (from 0.721 at visit 2 to 0.277 at visit 9) below the established cut-off (0.343) of the CKD model. Irrespective of the values before Irbesartan intake, the classification factors were decreasing during Irbesartan treatment in all patients except one (see **figure **3B). This patient progressed to DN several years after the end of the study, none of the eleven patients developed macroalbuminuria during the two year study period. In the urine samples of the eleven patients treated with placebo, a non significant (p = 0.1016) increase (indicating a change towards "chronic kidney disease") of the median classification factor (see **figure **3C) from -0.104 at visit 2 to 0.188 at visit 9 could be observed. Although many patients of the placebo-group scored lower than those of the Irbesartan-group at baseline before treatment (see **figure **3B and 3D), the classification factor of most placebo-treated patients was higher after two years, as expected for progressing disease.

We subsequently investigated which of the 273 biomarkers that were found significantly associated with CKD undergo significant changes upon Irbesartan treatment. Eighteen of these CKD markers showed significant differences (p < 0.05) in urine of patients before and after 2-year treatment with Irbesartan (see **table **1). Of these 18, 11 changed towards "normal controls", indicating possible benefit of therapy. Seven changes towards "chronic kidney disease", possibly indicating progression of pathophysiological changes over time that is not affected by therapy. We also investigated the 273 biomarkers in the placebo group. Here, we found 7 CKD markers which show significant differences within the 2-year treatment. Of these 7 peptides, all changed toward "disease". In total, 23 urinary CKD markers showed significant changes over the period of two years, either in the patients of the Irbesartan group or in the placebo group, 2 were significant in both groups. These two CKD markers, both collagen alpha-1 fragments (see **table **1, bold letters), showed significant change towards "healthy" in the Irbesartan group and opposite regulation in the placebo group over the period of two years. While the amount of these two collagen fragments increased significantly in the ARB group (indicating an improvement towards "healthy"), their abundance was significantly decreased after 2 years of placebo treatment, indicating further progression of chronic kidney disease.

**Table 1:** Significance analysis of CKD markers in urine of microalbuminuric patients before and after two year treatment

CKD marker	Sequence	Peptide name	Wilcoxon p-value (Irbesaran treatment)	Wilcoxon p-value (Placebo intake)	Irbesartan treatment	Placebo intake
2505	SpGEAGRpG	Collagen alpha-1 (I) chain [522-530]	1.58E-02	n.s.	↑	-
3508	GPpGPpGPpG	Collagen alpha-1 (I) chain [145-154]	1.58E-02	n.s.	↑	-
11982	YQTNKAKH	Cystatin-B [85-92]	2.55E-03	n.s.	↓	-
**13342**	**ApGDKGESGPS**	**Collagen alpha-1 (I) chain [777-787]**	**4.46E-02**	**8.33E-03**	↑	↓
14906	MGPRGPpGPpG	Collagen alpha-1 (I) chain [217-227]	n.s.	1.94E-02	-	↓
15800	GEYKFQNAL	Serum albumin [423-431]	1.53E-03	n.s.	↑	-
17694	ApGDRGEpGPp	Collagen alpha-1 (I) chain [798-808]	5.39E-05	n.s.	↑	-
**24117**	**SpGPDGKTGPPGp**	**Collagen alpha-1 (I) chain [546-558]**	**3.02E-02**	**1.58E-02**	↑	↓
24958	GPpGPDGNKGEpG	Collagen alpha-2 (I) chain [613-625]	1.84E-02	n.s.	↑	-
25053	GPpGEAGKpGEQG	Collagen alpha-1 (I) chain [650-662]	1.25E-03	n.s.	↑	-
28747	SpGERGETGPpGP	Collagen alpha-1 (III) chain [796-808]	4.10E-03	n.s.	↑	-
38780	GLpGTGGPpGENGKpG	Collagen alpha-1 (III) chain [642-657]	n.s.	2.62E-02	-	↓
55523	SpGSNGApGQRGEpGPQG	Collagen alpha-1 (III) chain [358-375]	n.s.	4.47E-02	-	↓
61573	DEAGSEADHEGTHSTKR	Fibrinogen alpha chain [605-621]	1.92E-02	n.s.	↑	-
73177	DAGApGAPGGKGDAGApGERGPpG	Collagen alpha-1 (III) chain [664-687]	7.81E-03	n.s.	↓	-
73697	GNSGEpGApGSKGDTGAKGEPGp	Collagen alpha-1 (I) chain [431-453]	n.s.	3.51E-02	-	↓
78332	AGPpGEAGKpGEQGVpGDLGAPGP	Collagen alpha-1 (I) chain [646-669]	1.04E-02	n.s.	↓	-
81196	NGApGNDGAkGDAGApGAPGSQGApG	Collagen alpha-1 (I) chain [700-725]	2.33E-02	n.s.	↓	-
82026	GNSGEpGApGSKGDTGAKGEpGPVG	Collagen alpha-1 (I) chain [431-455]	2.71E-02	n.s.	↓	-
94308	TGPIGPpGPAGApGDKGESGPSGPAGPTG	Collagen alpha-1 (I) chain [766-794]	1.30E-02	n.s.	↓	-
96370	LmIEQNTKSPLFMGKVVNPTQK	Alpha-1-antitrypsin [397-418]	4.47E-02	n.s.	↑	-
118224	ESGREGApGAEGSpGRDGSpGAKGDRGETGPA	Collagen alpha-1 (I) chain [1011-1042]	n.s.	6.94E-03	-	↓
143947	DQGPVGRTGEVGAVGPpGFAGEKGPSGEAGTAGPpGTpGPQG	Collagen alpha-2 (I) chain [824-865]	1.59E-03	n.s.	↓	-

Wilcoxon p-values (p < 0.05) of CKD markers, which show changes in the comparison of patients urine before and after longterm treatment are listed. In addition, sequences and peptide names of the significant markers are shown (p = hydroxyproline, k = hydroxylysine, m = oxidation of methionine). In the last three columns the regulation of those markers is depicted. Arrow upwards indicates significant change towards "healthy"; arrow pointing down indicates significant change towards "CKD".

To obtain information on additional changes in the urinary proteome associated with Irbesatan treatment beyond those observed for the previously defined CKD biomarkers, we examined the data on all sequenced peptides [40,52] for significant changes between baseline and 2-year treatment (in each group; Irbesartan and placebo). We could not identify additional biomarkers, which revealed significant changes between baseline and 2-year treatment.

## Discussion

In the IRMA2 study a 300 mg daily dose of the angiotensin II receptor blocker Irbesartan significantly reduced albuminuria compared to placebo [6]. Using CE-MS analysis of urine in all available samples (a subset of 22 of these patients) we were able to demonstrate a persistent and significant changes of the previously established proteomic CKD classifier [20] towards "healthy". After long-term renoprotective treatment with Irbesartan. Furthermore, the proteomic analysis of placebo treated patients showed a slight, yet not significant, increase of this classifier. This increase likely reflects disease progression in the absence of appropriate therapy, like blocking the renin angiotensin system demonstrated to protect against development of diabetic nephropathy.

We have previously reported that collagen fragments are reduced in patients with diabetic nephropathy [19]. After confirmation in additional samples, we generated the hypothesis that this reduction in urinary collagen fragments may be an indicator of attenuated collagen breakdown, resulting in fibrosis [53]. The results presented here further indicate that this process may be positively influenced by ARB treatment, resulting in an increase in urinary collagen fragments, likely reflecting an increase of proteolysis towards normal ("healthy") physiological levels. It is tempting to speculate that the urinary proteomic changes observed here may be a consequence of an actual change in renal pathophysiology, and not merely a consequence of the changes in urine protein concentration. To substantiate this hypothesis, analysis of longitudinal samples on a larger cohort will be undertaken.

As we also could show recently, the collagen fragments have similar quality as biomarkers in both, 24 h and spot urine [49]. This is to be expected since their secretion into urine does not appear to change significantly during the day (Mischak, unpublished), and the concentration of these biomarkers is assessed in reference to internal standards, in a similar way as albumin/creatinine ratio.

The changes in the urinary proteome reported here were observed employing a biomarker pattern that is associated with CKD in general, not restricted to diabetic nephropathy. This observation indicates that analysis of changes in the urinary proteome may also be useful in evaluation of treatments for other forms of kidney disease. Of note, drug-induced changes in the urinary proteome indicating benefit of therapy were recently reported for ANCA-associated vasculitis [21]. While the data currently available cannot clarify this issue, further analysis of urine samples from other therapeutic trials involving different drugs and other diseases (glomerulosclerosis and IgA Nephropathy) are planned. These may help to further support this hypothesis.

A shortcoming of the study reported here is the relatively low number of patients included. Unfortunately, no additional samples are available from the IRMA2 trial, hence this cannot be improved upon. However, the results were very consistent within each group. Even more relevant, we demonstrate on a very low number of only 11 treated and 11 untreated subjects, that ARB treatment does have a statistically significant positive effect, based on the proteomic CKD biomarker pattern, hence we feel that the report is in agreement with the recently published guidelines for proteomic biomarkers [54]. While we cannot exclude the presence of other confounders or underlying bias, we have no indication that confounders like e.g. drugs or infectious diseases at the time of sampling had a significance impact.

The results highlight an advantage of the urinary proteome analysis: a small number of subjects included in a trial may be sufficient to reveal significant effects of drug treatment, based on a classifier that serves as a surrogate marker. While such data can currently not replace hard endpoints like ESRD, they may serve to give guidance, e.g. for the decision if a drug may be likely to exert a positive influence on disease/disease progression.

## Conclusion

The data introduce urinary proteome analysis as a novel method not only for assessment of new drugs and therapeutic regimens in CKD, but also for the treatment monitoring of patients on renoprotective drugs. Furthermore, the data strengthen the hypothesis that collagens play an important role in the development of diabetic nephropathy (see also [53]) and that collagen turnover may be a highly suitable target for diagnosis and novel therapeutic approaches of this disease.

The proteomic biomarker pattern employed here (the CKD-273 pattern [20]) may well be a superior surrogate in comparison to the frequently used assessment of urinary albumin. To test this hypothesis, UAE and proteomic patterns from samples of longitudinal studies that reach hard endpoints have to be compared.

## Abbreviations

ARB: angiotensin II receptor blocker; CE-MS: capillary electrophoresis couples to mass spectrometry; CKD: chronic kidney disease; ESRD: end-stage renal disease; UAE: urinary albumin excretion; RAAS: renin-angiotensin-aldosterone system; IRMA-2: Irbesartan in Patients with Type 2 Diabetes and Microalbuminuria Study

## Competing interests

Harald Mischak is co-founder and a co-owner of mosaiques diagnostics & therapeutics AG, (Hannover, Germany). Petra Zürbig is an employee of mosaiques diagnostics GmbH. Peter Rossing has received speakers honorarium from Novartis, Sanofi-Aventis, Boehringer Ingelheim, and MSD, and research grants from Novartis. Hans-Henrik Parving has received speakers honorarium from Novartis and consulting fees from Novartis.

## Authors' contributions

SA participated in the design of the study and performed the statistical analysis. PZ and HM performed the CE-MS analysis and data evaluation. H-HP and PR conceived of the study, and participated in its design and coordination. All authors were involved in drafting the manuscript, have read and approved the final manuscript.

## Pre-publication history

The pre-publication history for this paper can be accessed here:

http://www.biomedcentral.com/1471-2369/11/29/prepub

## Supplementary Material

Additional file 1 **Raw data and additional information**. Table consists of 4 different spreadsheets called patients data, classification factor, polypeptides, and patient's raw data. **Patients data. **This table lists information of each patient, including patients IDs and treatment. Furthermore urinary albumin concentration, eGFR, and blood pressure are given at baseline and after two years. **Classification factors. **Table show the classification factors of all measured urine samples, including patients IDs, sample ID, and treatment. **Polypeptides**. Table listing 2,044 different peptides/proteins (Protein ID) detected, their calibrated molecular mass [Da], and normalized CE migration time [min]. Furthermore, sequence information is given, if available. **Patient's raw data**. Tables in pivot format show the CE-MS raw data of the 44 samples in the database. The protein IDs of all peptides are given in the first column named "Protein ID"; the unique patients IDs constitute the first row. The MS data from each sample are reflected in one column. The number in each cell represents the calibrated amplitude of the mass spectrometric signal of each peptide/protein detected in the sample. Click here for file
